# Supplementary material for: Using Extreme Value Statistics to Reconceptualize Psychopathology as Extreme Deviations From a Normative Reference Model
Source: Hum Brain Mapp. 2025 Jul 19;46(11):e70281. doi: 10.1002/hbm.70281 (PMC12275014; doi:10.1002/hbm.70281)
Supplement: Supplementary file 1 — Appendix S1. Supplementary Information. [file HBM-46-e70281-s001.docx]

**Supplementary Methods**

**Sample**

For the distribution of the age range and the exact number of participants in our dataset of the image derived phenotypes (IDPS), see Figure 1.


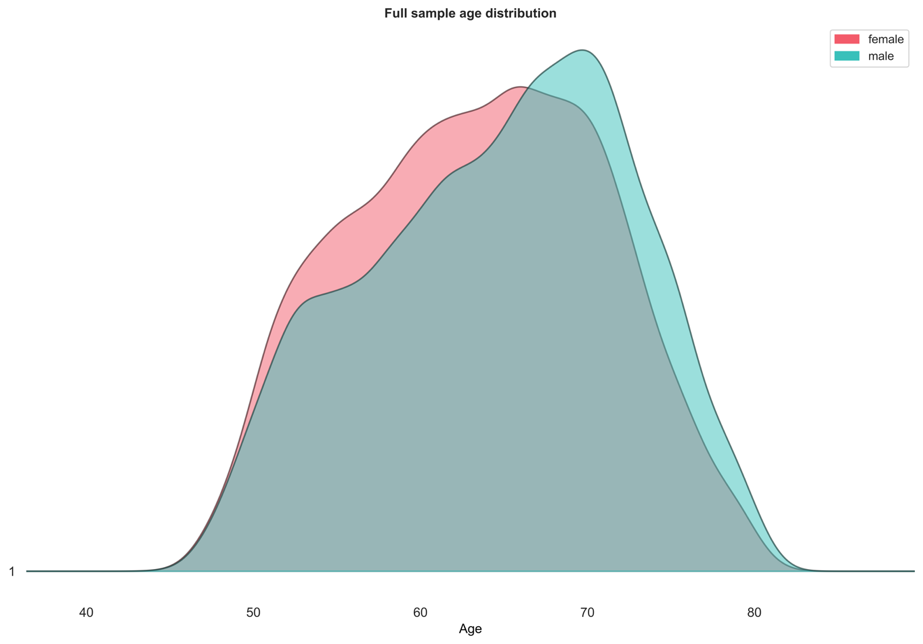

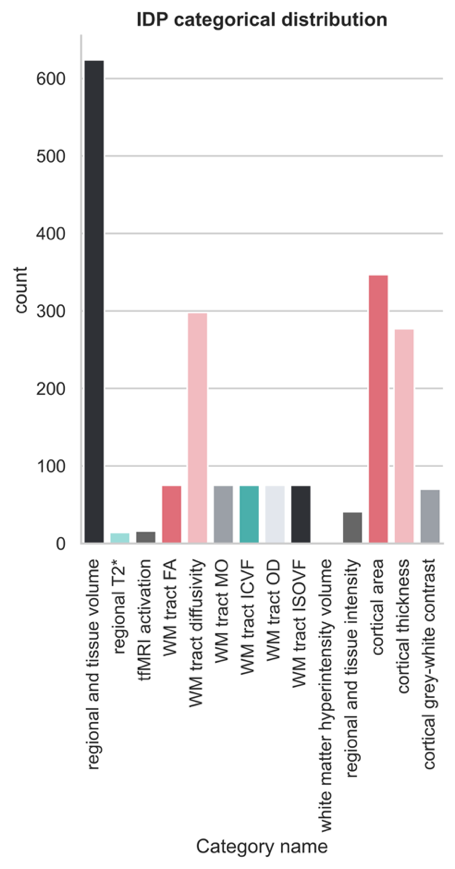


Figure 1 – On the left - showing the age and sex distribution (53% females) of the IDPs used in this study from the UK biobank. On the right - showing the distribution of the different categories present in the UK Biobank dataset. Abbreviations used in the plot: Image derived phenotype (IDP), White matter (WM), Fractional Anisotropy (FA), mode of diffusivity (MO), isotropic or free water volume fraction (ISOVF).

**Normative model formulation**

We estimated all the normative models using Python 3.8.3 and the PCNtoolkit version 0.20. We performed a normative model with the Bayesian linear regression model (BLR) and likelihood warping. For a full explanation and details of the mathematical framework behind this model, see [1]. Here, we will give a short overview of the mathematics behind the method. We define $\boldsymbol{y}=\left( y_{nd} \right)\in\mathbb{R}^{N\times D}$ with $y_{nd}$ the $d$-th IDP of the $n$-th subject. The covariates are collected into one matrix $\boldsymbol{x}=\left( x_{nm} \right)\in\mathbb{R}^{N\times M}$, where $x_{nm}$ is the $m$-th covariate of the $n$-th subject:

$\boldsymbol{y}=\left[ \begin{matrix} y_{11} & y_{12} & \ldots& y_{1d} \\ y_{21} & y_{22} & \ldots& y_{2d} \\ \vdots& \vdots& \ddots& \vdots\\ y_{n1} & y_{n2} & \ldots& y_{nd} \end{matrix} \right]$ and $\boldsymbol{x}=\left[ \begin{matrix} x_{11} & x_{12} & \ldots& x_{1m} \\ x_{21} & x_{22} & \ldots& x_{2m} \\ \vdots& \vdots& \ddots& \vdots\\ x_{n1} & x_{n2} & \ldots& x_{nm} \end{matrix} \right]$

We used the covariates age, sex, and site, with site referring to scanner site, as reported in [2]. Here the sites are denoted by $s\in\{1, \ldots, S\}$ with all subjects having the same sites. For each IDP we fitted a separate model. To keep the notation concise, we will concentrate on one specific IDP labelled $d$ and drop the subscript. Thus, for every IDP we denote $\boldsymbol{y}=\left( y_{1},\ldots, y_{N} \right)^{T}$ and take the set of independent variables $\boldsymbol{x}_{n}=\left( x_{n1},\ldots,x_{nM} \right)^{T}$. For every subject, we specified the model as follows:

${\varphi(y}_{n})=\boldsymbol{w}^{T}\phi\left( \boldsymbol{x}_{n} \right)+\epsilon_{s}$ (1)

Where, $\boldsymbol{w}^{T}$ is the estimated vector of weights and $\phi\left( \boldsymbol{x} \right)$ is a basis expansion of the covariate vector $\boldsymbol{x}_{\boldsymbol{n}}$. In our case, a cubic B-spline basis expansion with five evenly spaced knots was chosen. Empirically, this was enough to capture the curvature in space caused by the age covariate.$\epsilon\mathcal{=N}(0,\beta^{-1})$ is a Gaussian noise distribution with mean zero and noise precision term $\beta$ (the reciprocal of the variance). $\varphi\left( y_{n} \right)$ is a likelihood warping function used to accommodate non-Gaussianity of the residuals of the data in the original space. For the likelihood warping a SinhArcsinh function was employed:

${\varphi(y_{n}, \boldsymbol{\gamma})}_{SinhArcsinh}=sinh(c*\mathrm{arcsinh} \left( y_{n} \right)-e)$ (2)

With $\boldsymbol{\gamma=(}c,e)$ the identified parameters for the warping function. This method has been shown to be able to model Gaussian as well as non-Gaussian distributions [1]. If non-Gaussianity is present in the data, there are other techniques besides likelihood warping that one can consider. A pre-transformation of the dependent variable, like a Box-Cox or log-transform, is one illustration. Finding the right transformation beforehand that is optimal across various datasets, however, can be quite difficult. The warped BLR model thus has the additional benefit of eliminating the extra step of selecting an appropriate transformation for each dataset by incorporating this in the model through likelihood warping [1], [3]. We captured the site variation using a fixed-effects model, according to [4]. We performed the optimization using Powell's conjugate direction method by minimizing the negative log-likelihood. Afterward, the *z*-scores were calculated in the warped space for each subject, *n*, and IDP, *d*, in the test set as:

$z_{nd}=\frac{y_{nd}-\hat{y}_{nd}}{\sqrt{\sigma_{d}^{2}+{(\sigma_{*}^{2})}_{d}}}$ (3)

Where, $y_{nd}$ is the true response*,* $\hat{y}_{nd}$ is the predicted mean, $\sigma_{d}^{2}$ is the estimated noise variance (reflecting uncertainty in the data), and ${(\sigma_{*}^{2})}_{d}$ is the variance attributed to modeling uncertainty, for the full derivations see [1], [5]. We evaluated the model fits according to several model criteria: explained variance ($R^{2}$), mean squared log-loss (MSLL), and skew and kurtosis. Together, these criteria allowed us to assess the central tendency, performance of the warping function, as well as, overall model fit. Afterward, we used the *z*-scores to estimate the extreme value distributions.

**Additional details about extreme value statistics**

The boxes below provide further details about the generalized extreme value (GEV) distribution and generalized Pareto distribution (GPD).

**The Generalized Extreme Value Distribution**

Let $Z_{1},Z_{2},\ldots, Z_{n}$ be a sequence of independent random variables with common distribution function $F$, and:

$$M_{n}=\max\left( Z_{1},\ldots, Z_{n} \right) (4)$$

**Theorem 1:** If there exist sequences of constants {$a_{n}>0$} and {$b_{n}$} such that the probability (P):

$P \left\{ \left( \frac{M_{n}-b_{n}}{a_{n}} \right)\leq z \right\}\to G\left( z \right), n\to\infty$ (5)

Where $G$ is a non-degenerate distribution function, then $G$ belongs to one of the following families:

$I:G\left( z \right)=\exp\left\{ -\exp\left[ -\left( \frac{z-b}{a} \right) \right] \right\}, -\infty<z<\infty;$ (6)

$II:G\left( z \right)=\left\{ \begin{aligned} 0, \\ \exp\left\{ -\left( \frac{z-b}{a} \right)^{-\alpha} \right\}, \end{aligned} \right.\begin{matrix} z\leq b; \\ z>b; \end{matrix}$ (7)

$III:G\left( z \right)= \left\{ \begin{aligned} \exp\left\{ -\left[ -\left( \frac{z-b}{a} \right)^{\alpha} \right] \right\}, \\ 1, \end{aligned} \right. \begin{matrix} z<b; \\ z\geq b; \end{matrix}$ (8)

for parameters $a$ > 0, $b$and, in the case of families $II$ and $III$, $a>0$. The extreme value distributions consist of three categories, which are commonly referred to as the Gumbel, Fréchet, and Weibull families, known as types $I$, $II$, and $III$, respectively. Each family is characterized by a location parameter ($b$) and a scale parameter ($a$). The Frechet and Weibull families also have a shape parameter ($\alpha$). The Gumbel, Fréchet, and Weibull families can be merged into a unified family of models called the generalized extreme value (GEV) family of distributions.

**Theorem 1.1**: If there exist sequences of constants {$a_{n}>0$} and {$b_{n}$} such that:

$P \left\{ \frac{M_{n}-b_{n}}{a_{n}}\leq z \right\}\to G\left( z \right), n\to\infty$ (9)

For a non-degenerate distribution function $G$, then $G$ is a member of the GEV family:

$G\left( z \right)=\exp\left\{ -\left[ 1+\xi\left( \frac{z-\mu}{\sigma} \right) \right]^{-\frac{1}{\xi}} \right\}$ (10)

Defined on $\{z:1+\frac{\xi\left( z-\mu\right)}{\sigma}>0\}$, where $-\infty<\mu<\infty, \sigma>0$ and $-\infty<\xi<\infty$.

**The Generalized Pareto Distribution**

**Theorem 2:** Let $Z_{1},Z_{2},\ldots, Z_{n}$ be a sequence of independent random variables with common distribution function $F$, and:

$$M_{n}=\max\left( Z_{1},\ldots, Z_{n} \right) (11)$$

suppose that $F$ satisfies Theorem 1.1, so that for large $n$,

$P\{M_{n}\leq z\}\approx G(z)$(12)

Where

$G\left( z \right)=\exp\left\{ -\left[ 1+\xi\left( \frac{z-\mu}{\sigma} \right) \right]^{-\frac{1}{\xi}} \right\}$ (13)

for some $\mu, \sigma>0$ and $\xi$. Then, for a large enough threshold $k$, the distribution function of $(Z-k)$, conditional on $Z>k$, is approximately:

$H\left( y \right)=1-\left( 1+\frac{\xi y}{\tilde{\sigma}} \right)^{-\frac{1}{\xi}}$ (14)

Defined on $\{y:y>0 and \left( 1+\frac{\xi y}{\tilde{\sigma}} \right)>0\}$, where

$$\tilde{\sigma}=\sigma+\xi(k-\mu)$$

This family of distributions is called the generalized Pareto family.

**Supplementary Results**

**Bayesian Linear Regression model fit**

We evaluated the performance of the BLR model and the likelihood warping by looking at the explained variance, MSLL, skewness, and kurtosis, see Figure 2.


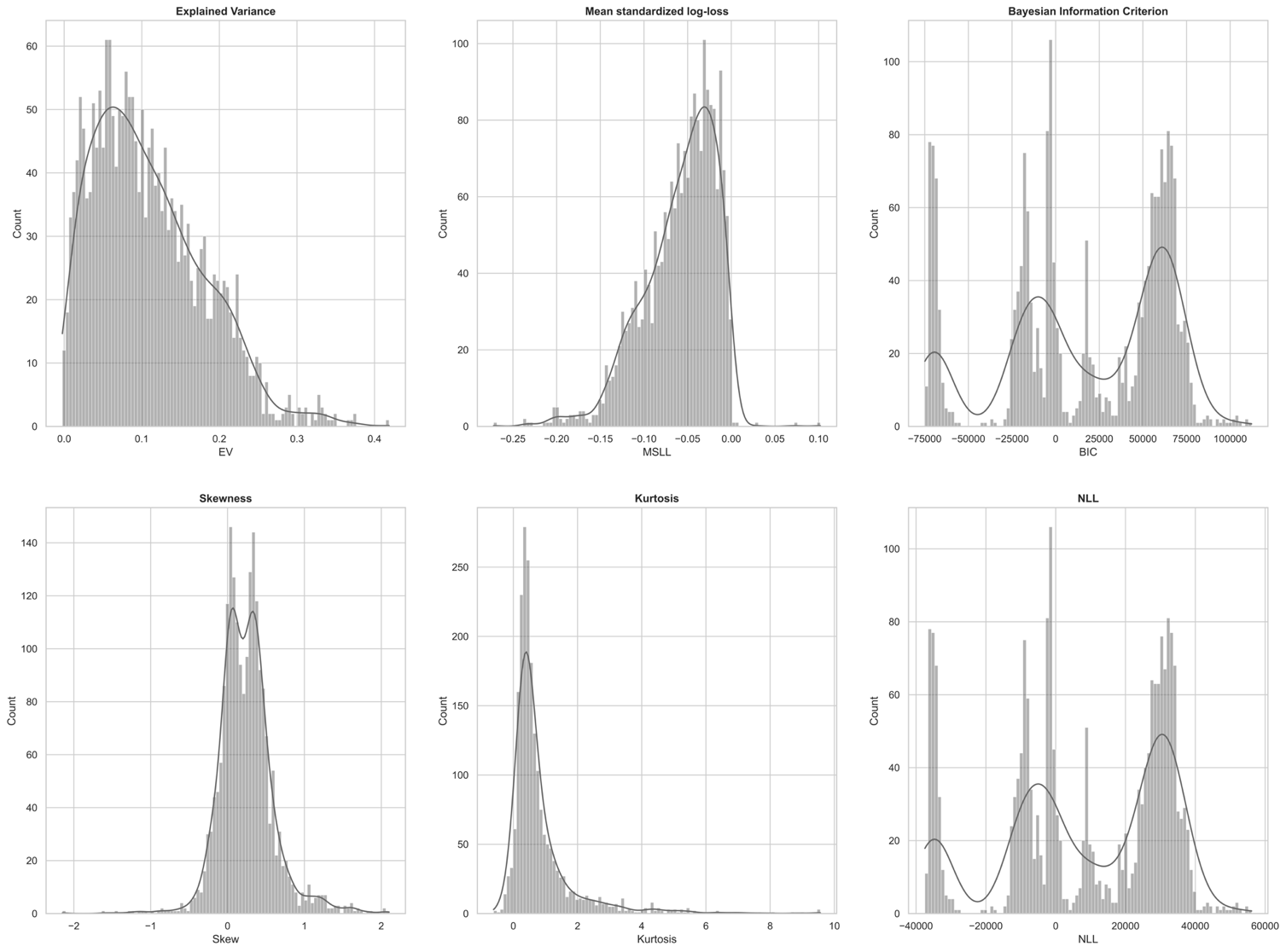


Figure 2 - Showing the performance measures for the different IDPs using a warped BLR model. In general, for an optimal model fit, the skew and kurtosis would both be distributed around zero.

Figure 3 - Displaying various threshold selection results for IDP 25397 – mean Orientation Dispersion (OD) in fornix on Fractional Anisotropy (FA) skeleton. A. illustrates the histogram of the z-scores, demonstrating different thresholds that could be employed in peaks over threshold method. B. displays the mean residual life plot, showing a relatively linear plot up to the 0.95 threshold, indicating that this could be a good threshold. C. and D. show the parameter threshold stability plot of the modified shape and modified scale parameters. The dots demonstrate the shape and modified scale parameters for the Generalized Pareto distribution (GPD) at different thresholds. The lines show the 95% confidence interval. An optimal threshold is chosen at the point where both parameters are relatively stable, in this case, around the 95th percentile.

Figure 4 - Displaying various threshold selection results for IDP 25699 - weighted-mean Orientation Dispersion (OD) in tract superior longitudinal fasciculus (right). A. illustrates the histogram of the z-scores, demonstrating different thresholds that could be employed in the peaks over threshold method. B. displays the mean residual life plot, showing a relatively linear plot up to the 0.95 threshold, indicating that this could be a good threshold. C. and D. show the parameter threshold stability plot of the modified shape and modified scale parameters. The dots demonstrate the shape and modified scale parameters for the Generalized Pareto distribution (GPD) at different thresholds. The lines show the 95% confidence interval. An optimal threshold is chosen at the point where both parameters are relatively stable, in this case, around the 95th percentile.

Figure 5 - Displaying various threshold selection results for IDP 25599 - Weighted-mean L2 in tract anterior thalamic radiation (right). A. illustrates the histogram of the z-scores, demonstrating different thresholds that could be employed in the peaks over threshold method. B. displays the mean residual life plot, showing a relatively linear plot up to the 0.95 threshold, indicating that this could be a good threshold. C. and D. show the parameter threshold stability plot of the modified shape and modified scale parameters. The dots demonstrate the shape and modified scale parameters for the Generalized Pareto distribution (GPD) at different thresholds. The lines show the 95% confidence interval. An optimal threshold is chosen at the point where both parameters are relatively stable, in this case, around the 95th percentile.

**Extreme value theory results**

In Figure 6, we show the data from two IDPs in the original space and after the marginal transformations have been applied. Furthermore, we show the histogram of the radius parameter ($r$) exceeding the 0.95 quantile threshold. It can be noted that the variables fully lie on their respective axis after the marginal transformation.

Figure 6 - Illustration of the applied marginal transformation to the IDP data. A. Scatterplot of two IDPs in their original dimensions. B. Scatterplot of the IDP data after transforming the marginal, such that the marginal is regularly varying with α=2. C. Histogram showing the r polar coordinate and the 0.95 largest components.

The number of participants exceeding the 0.95 quantile threshold was $n_{exc}=1960$ data. This data is then used to estimate the tail pairwise dependence matrix TPDM ($\hat{\Sigma_{\mathbf{z}}}$).

**Interpretation of extreme principal components**

We performed a standard eigendecomposition of $\hat{\Sigma}_{\boldsymbol{z}}$ or the TPDM to obtain the eigenvectors (i.e. extreme principal components (PCs)). Similar to covariance-PCA, we can look at the amount of scale that the first number of eigenvalues explains. In Figure 7, we show the correlation circle for the first two extreme PCs.


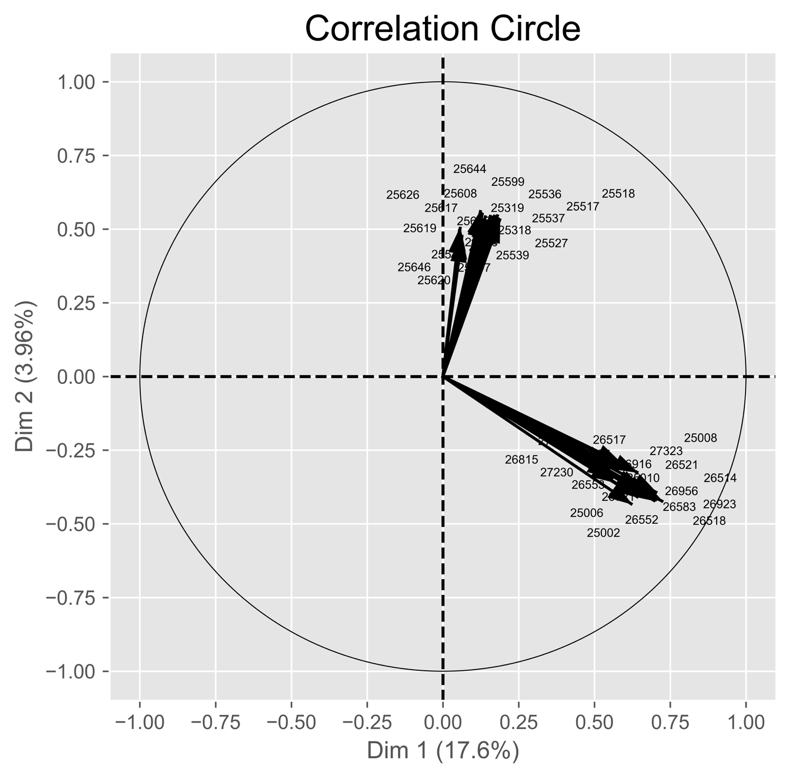


Figure 7 – Showing the correlation circle with the loadings of the top 40 contributing variables on the extreme principal components 1 and 2.

**Removed IDPs**

Since our objective was to demonstrate a new method, we removed a small number of IDPs, which the likelihood warping approach did not fit well. An example is shown below.


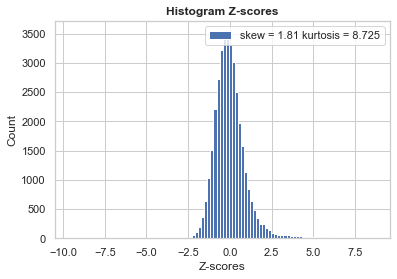

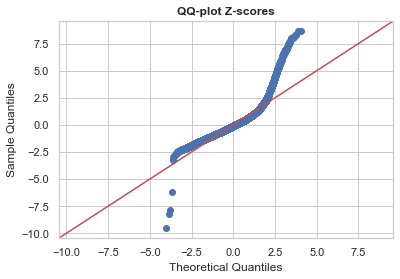


Figure 8 - Showing an example of a badly fitted IDP with a high kurtosis. In the analysis, we have removed IDPs with a kurtosis above 10 from further processing, excluding one IDP.

**Standard PCs correlation with behavior**

Here, we show the results for correlating standard PCs to the behavioral variables. Figure 11 presents the Manhattan plots of the p-values for the univariate Spearman correlation between the non image derived phenotypes (nIDPs) and the first two standard PCs.


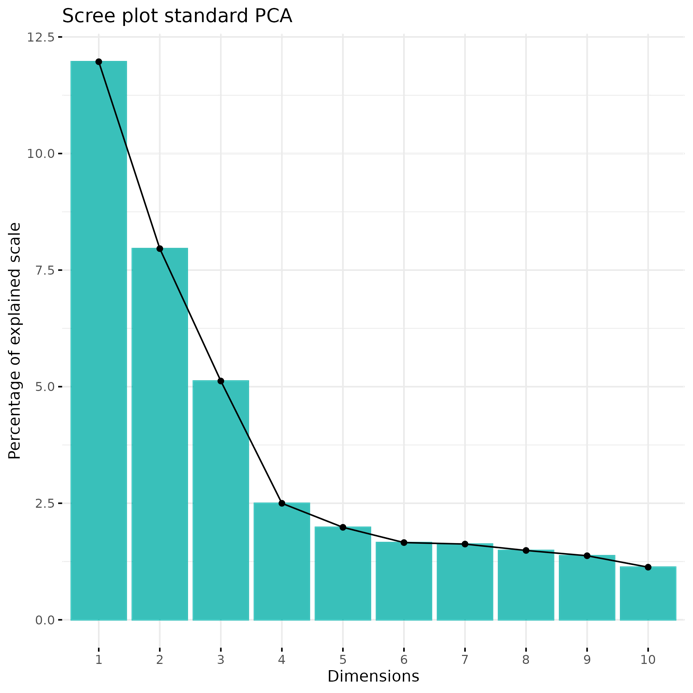

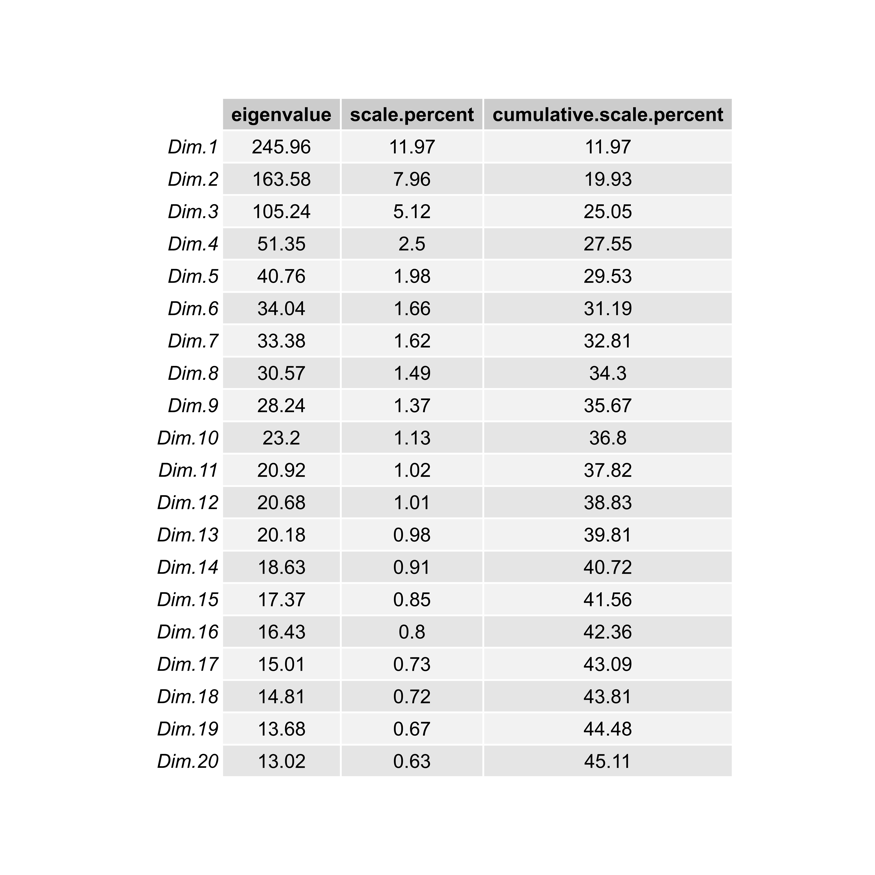


Figure 9 - Showing the scree plot of the standard PCA and table of scale values of the eigenvalue decomposition for the covariance matrix.


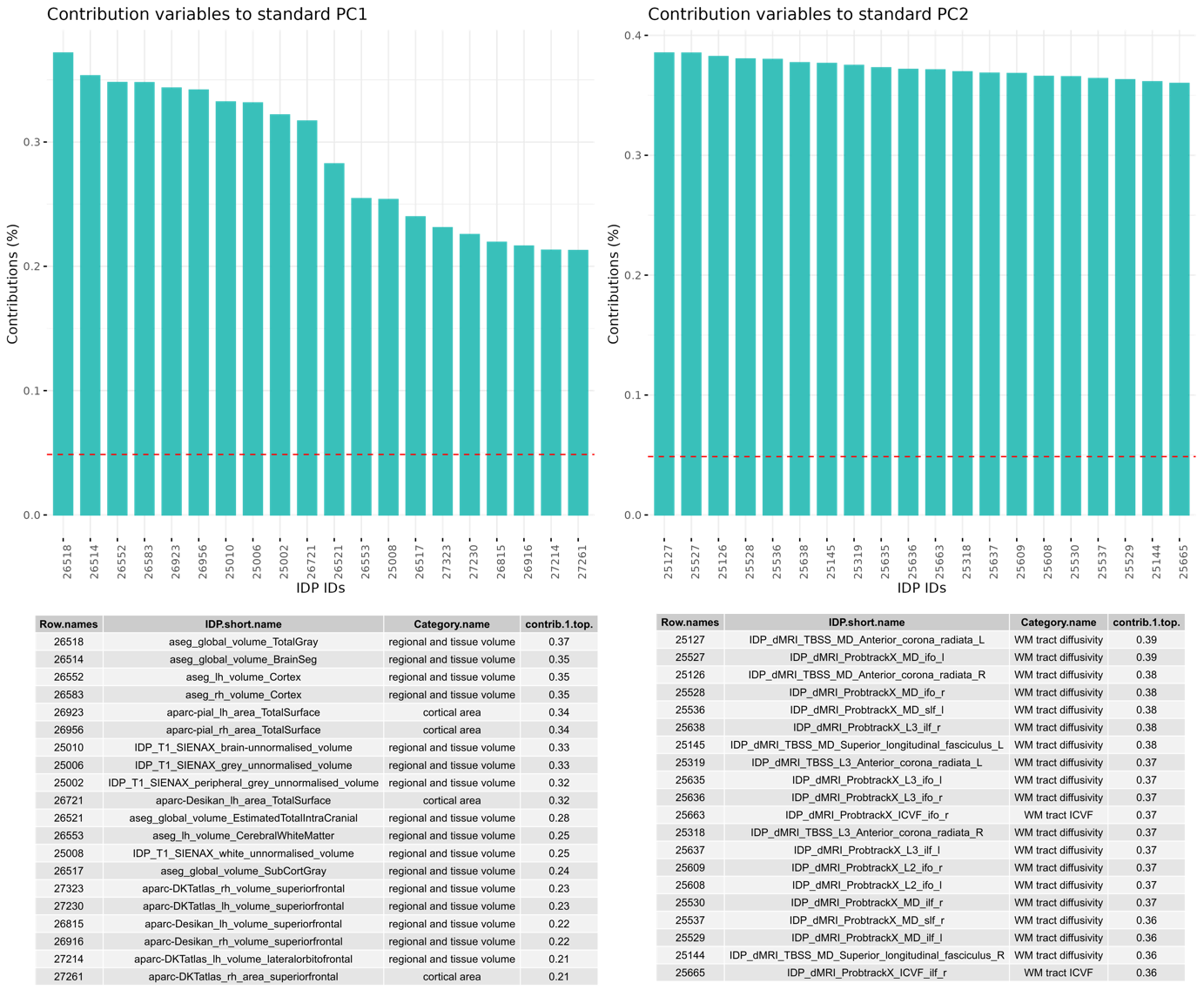


Figure 10 - List of the top 20 contributions of the different IDPs to the first and second standard PCs.

**
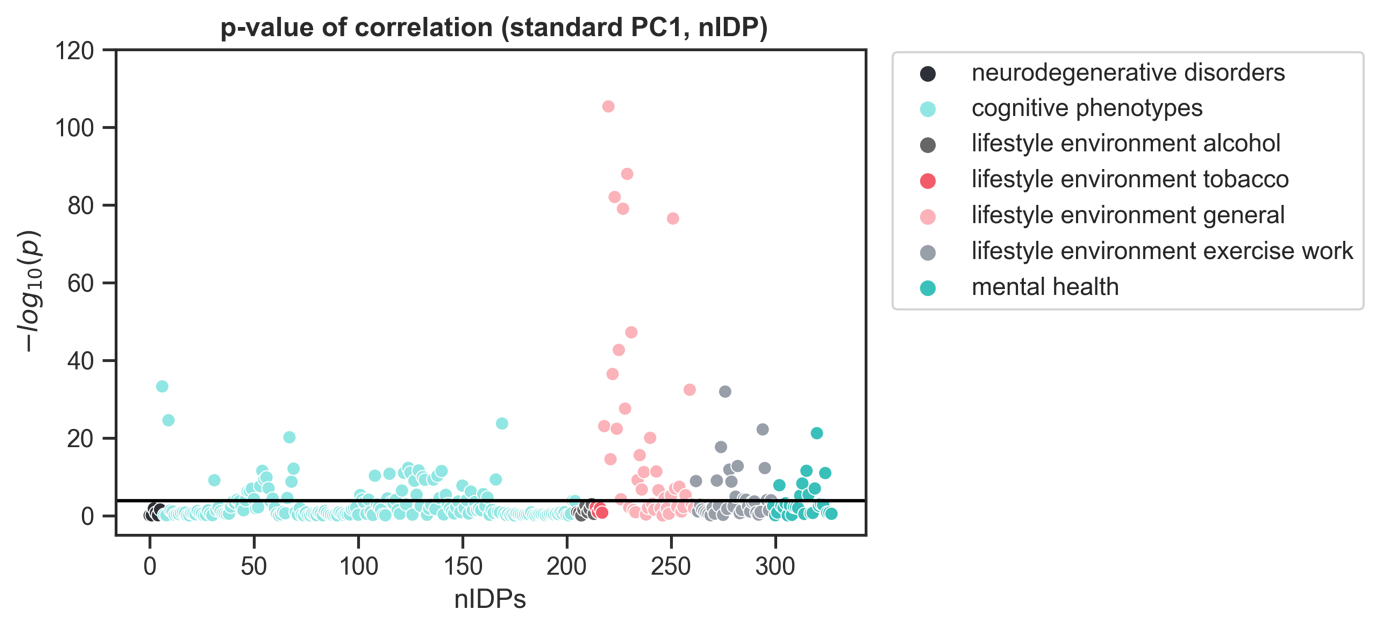
**

**
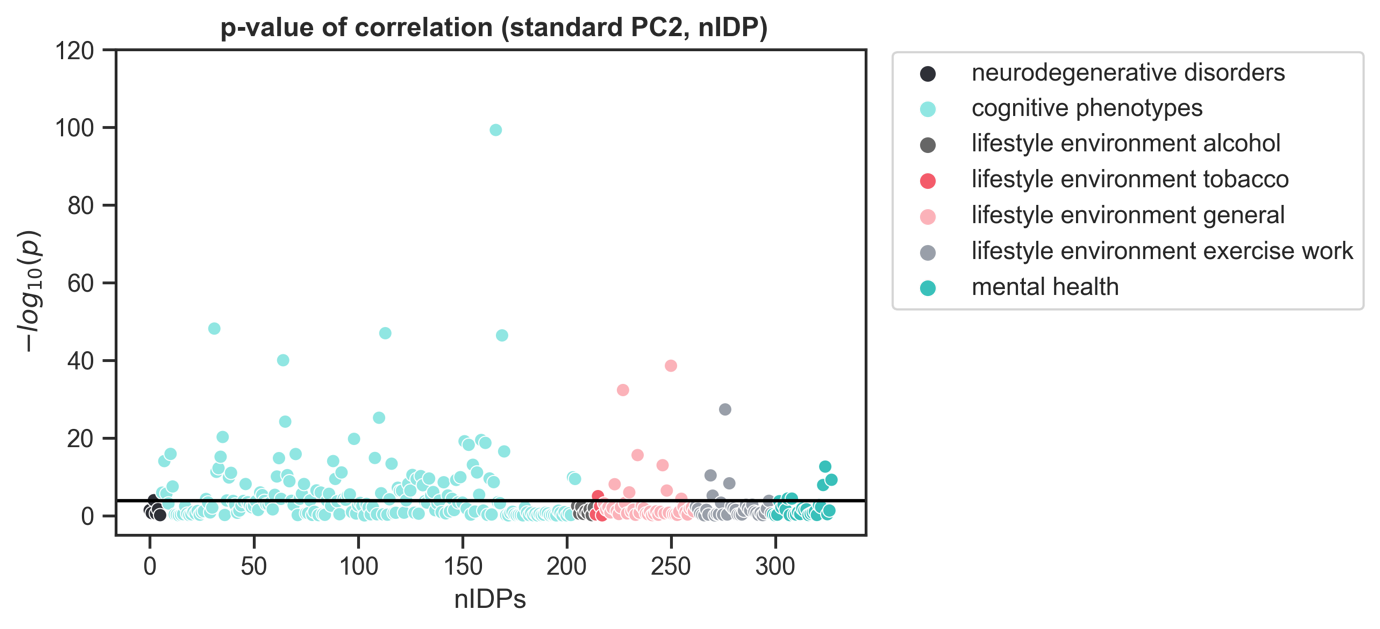
**

Figure 11 - Showing two Manhattan plots of the log p-values for the Spearman correlation between the nIDPs and the first two standard PCs. The black line demonstrates the Bonferroni-corrected p-value threshold. Thus, values passing this line indicate a significant PC-nIDP correlation.

**Univariate extreme count score correlation with behavior**

Here, we present the results of correlating the number of positive (sum(Z > 1.96)) and negative (sum(Z < -1.96)) deviations with behavioral variables. This approach aligns with previous studies that have examined correlations between behavior and deviation scores[2], [6]. Figure 12 shows Manhattan plots of the p-values for the univariate Spearman correlation between nIDPs and the first two standard PCs. Notably, many previously identified patterns are still observed with this simpler analysis. However, because this method relies on a basic count score, it is more challenging to determine which specific IDP primarily drives the multivariate effect.

**
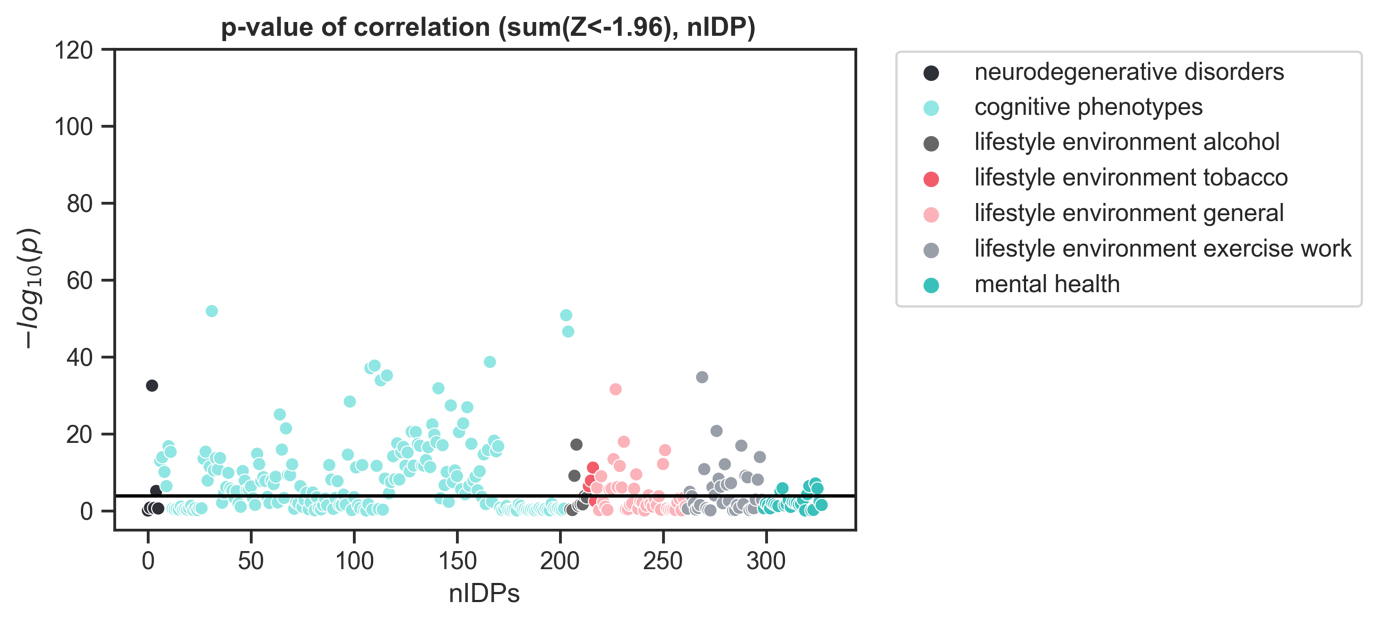
**

**
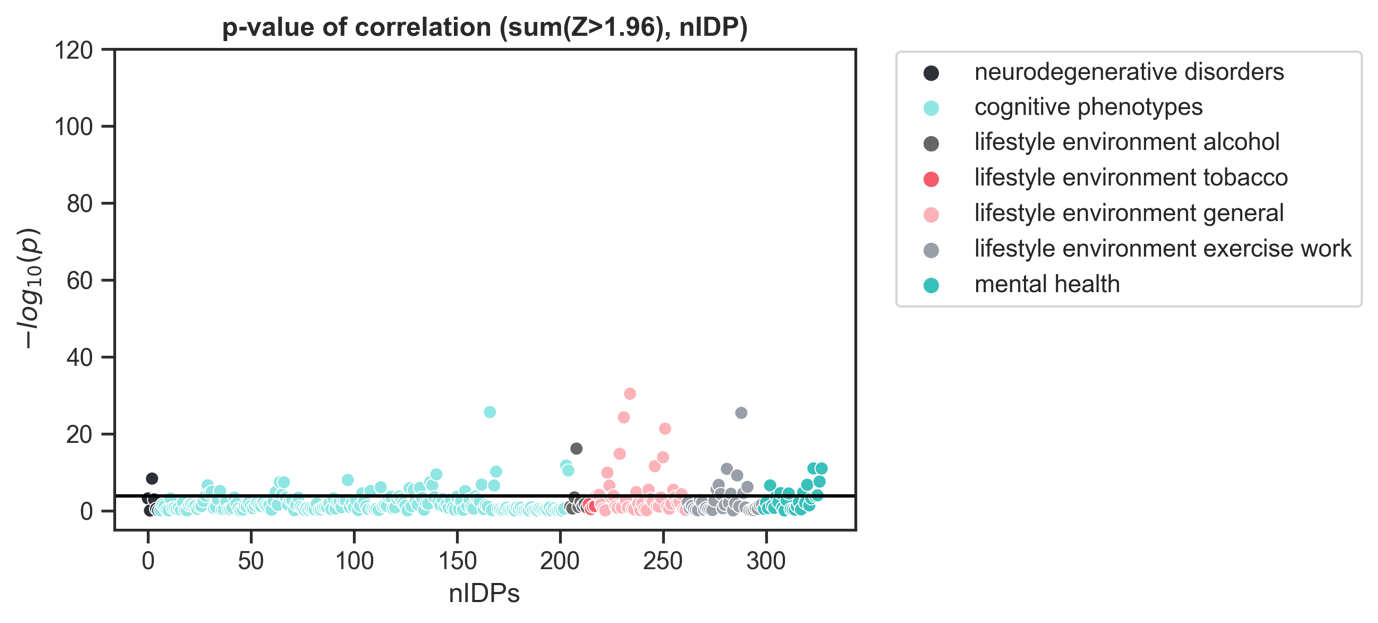
**

Figure 12 - Showing two Manhattan plots of the log p-values for the Spearman correlation between the nIDPs and the sum count of the negative deviations (Z<-1.96) and the positive deviations (Z>1.96). The black line demonstrates the Bonferroni-corrected p-value threshold. Thus, values passing this line indicate a significant PC-count-score correlation.

**Comparison effect sizes**

Figures 13, 14, and 15 show the effect sizes for the extreme PCA, standard PCA, and count score analysis. Specifically, we present the Spearman’s correlation coefficients (r) as a measure of the effect size, offering additional information on the direction of the correlation between the brain-derived measures and the nIDPs.


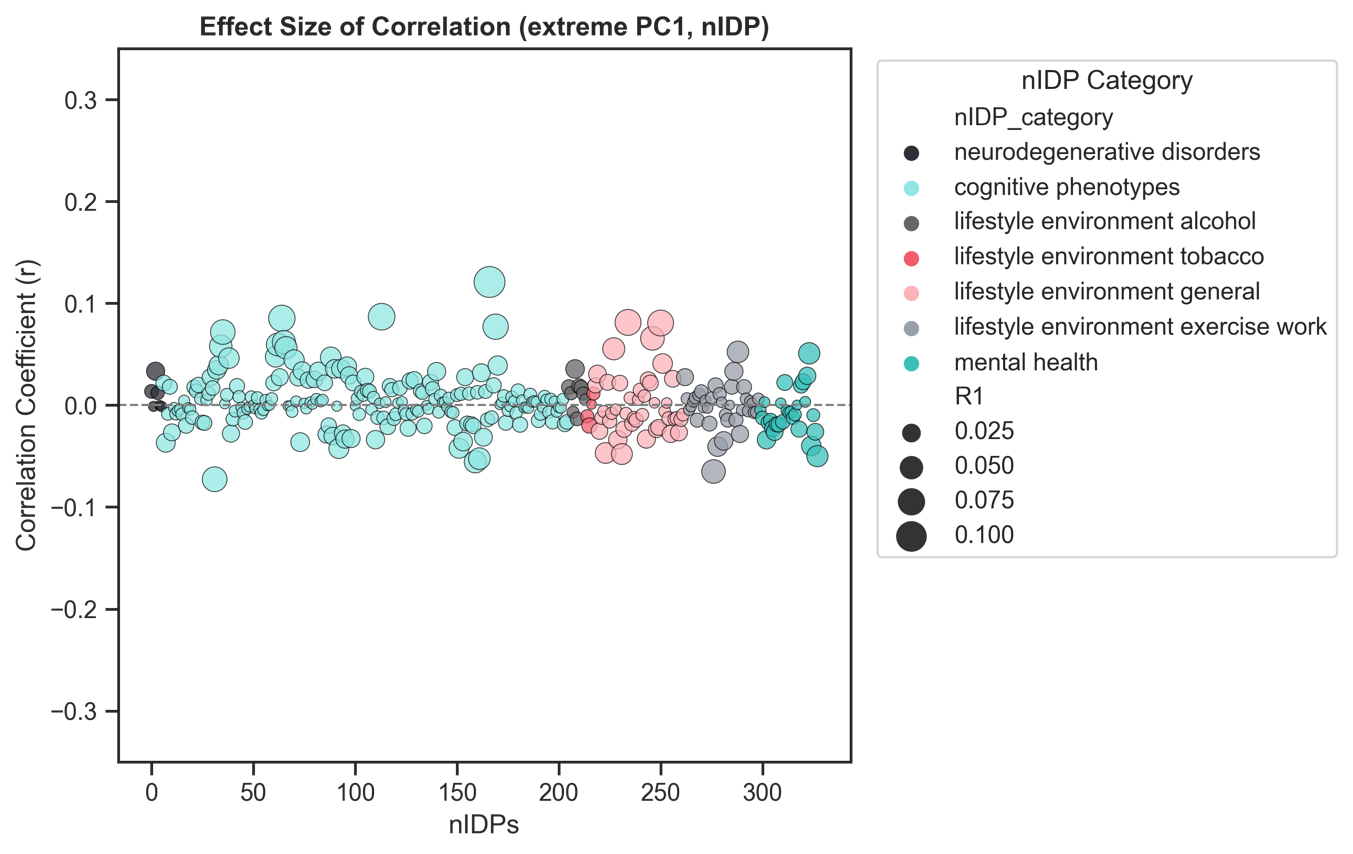


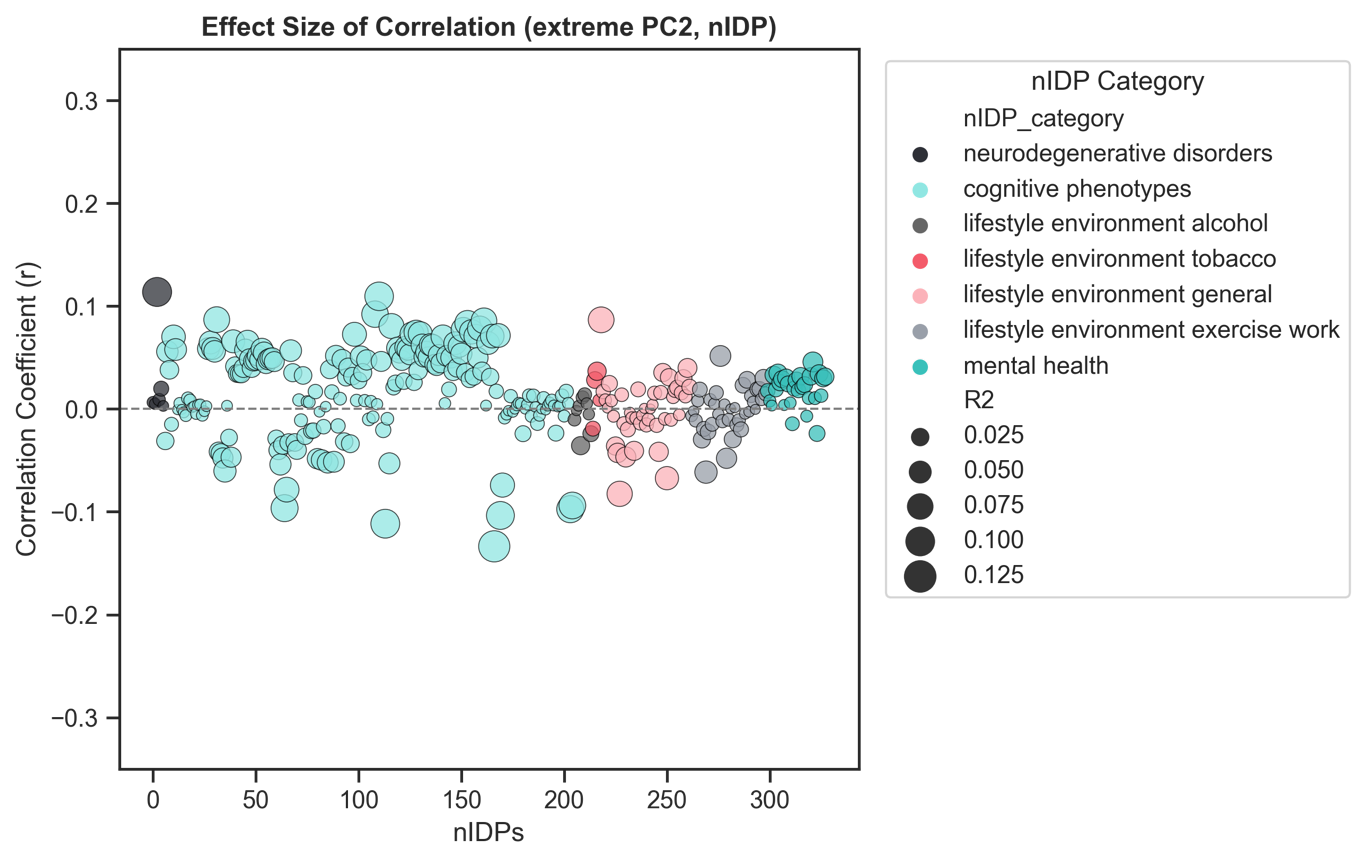


Figure 13 - Showing two Manhattan plots of effect size for the Spearman correlation between the nIDPs and the first two extreme PCs.


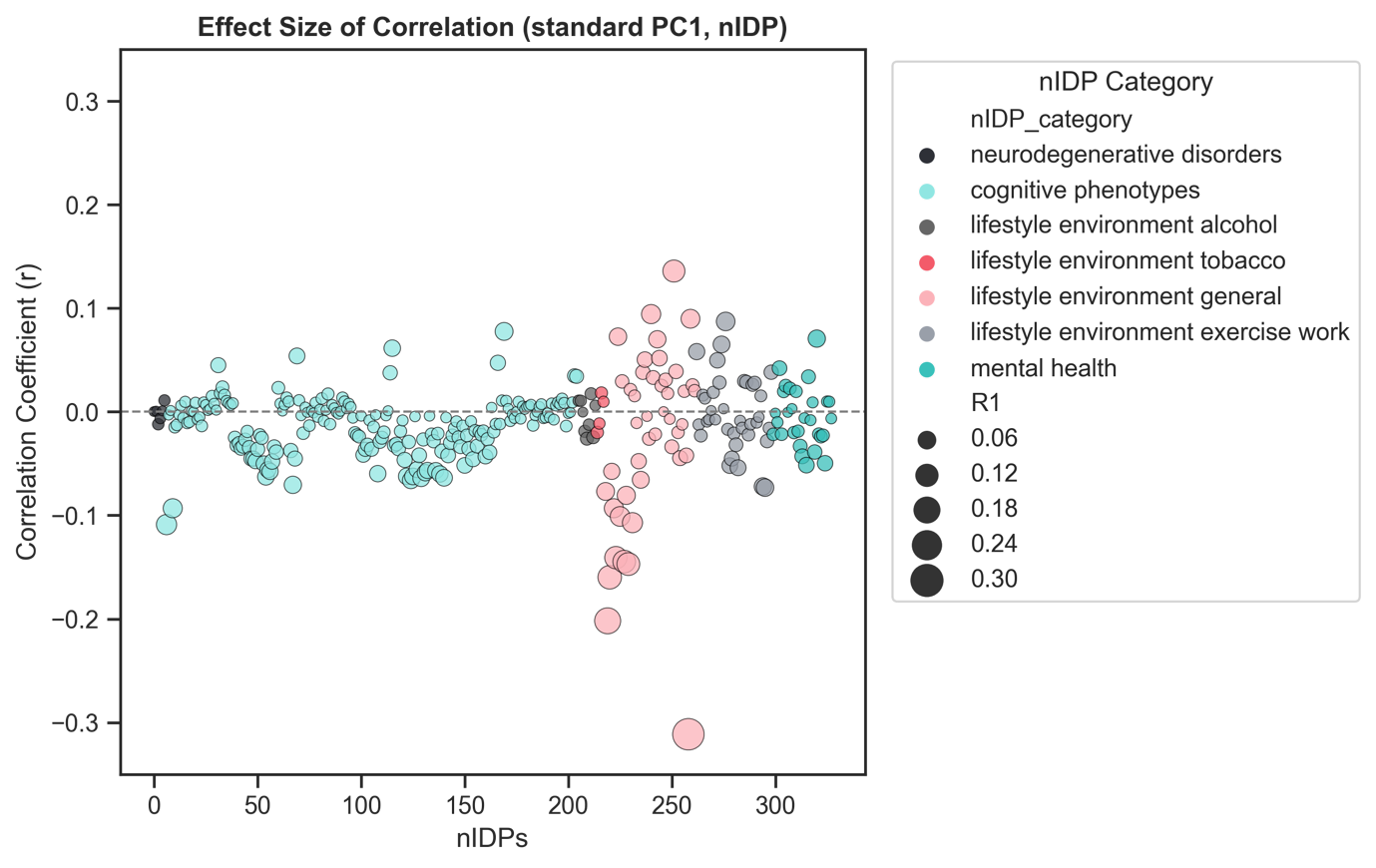


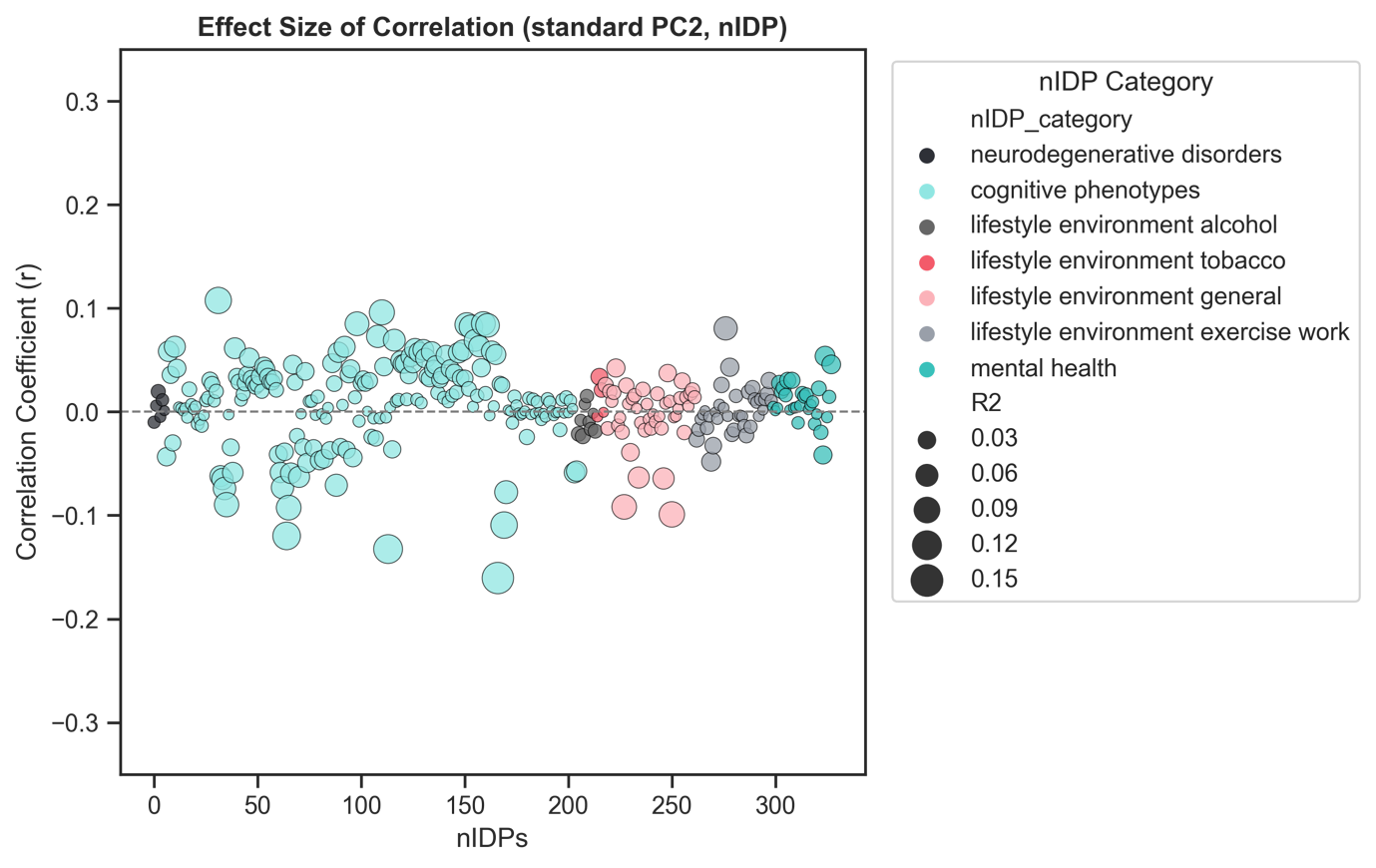


Figure 14 - Showing two Manhattan plots plots of effect size for the Spearman correlation between the nIDPs and the first two standard PCs.


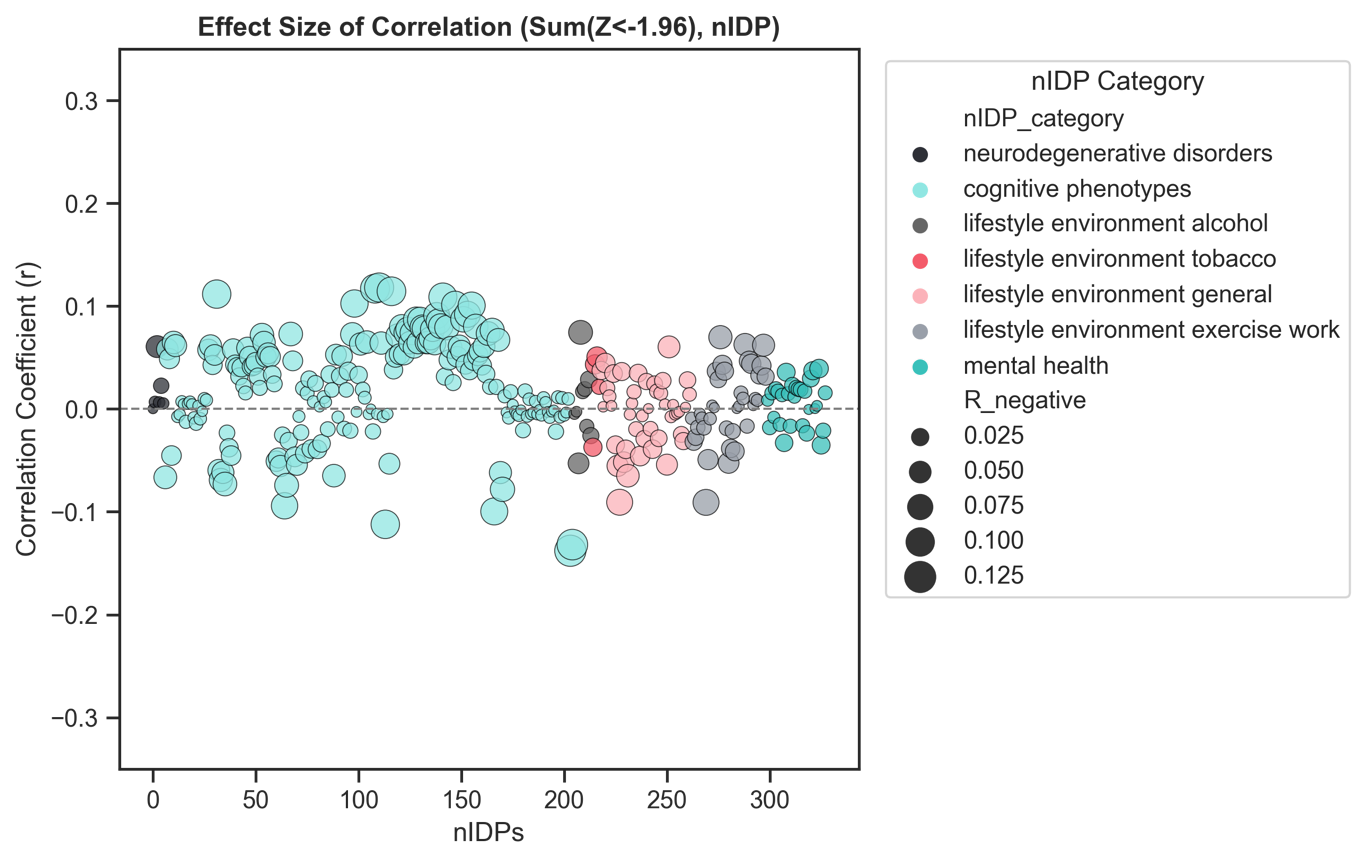


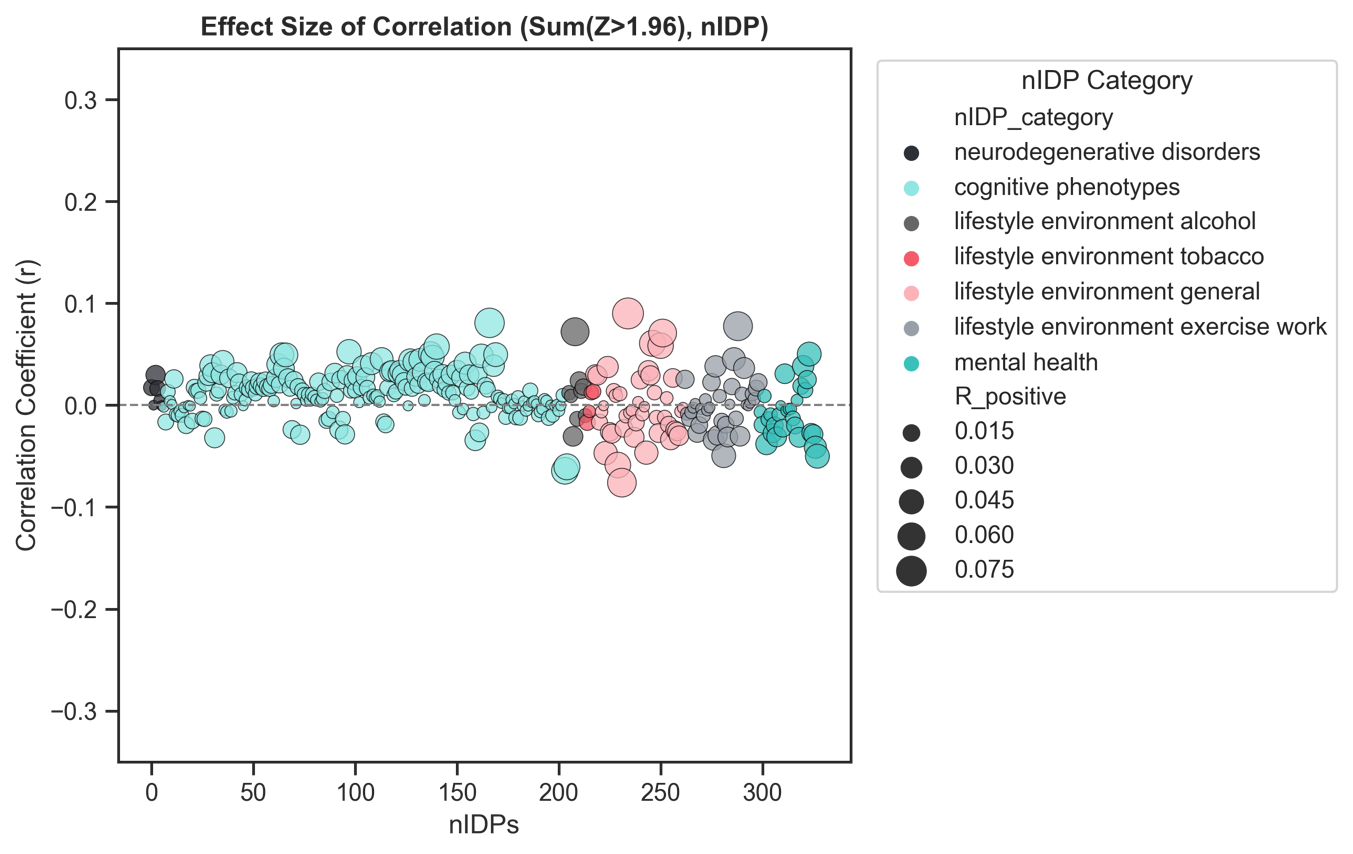


Figure 15 - Showing two Manhattan plots of effect size for the Spearman correlation between the nIDPs and the sum count of the negative deviations (Z<-1.96) and the positive deviations (Z>1.96).

| **UKB nIDP ID** | **-log_10_(p)** | **Description** |
| --- | --- | --- |
| ***Neurodegenerative disorders*** | | |
| *No hits* |  |  |
| **Cognition** |  |  |
| 20016-2.0 | 99.2 | Fluid intelligence score |
| 630-2.0 | 48.1 | Touchscreen duration |
| 6373-2.0 | 46.9 | Number of puzzles correctly solved |
| 20128-2.0 | 46.4 | Number of fluid intelligence questions attempted within time limit |
| 4282-2.0 | 40.0 | Maximum digits remembered correctly |
| 398-2.3 | 33.2 | Number of correct matches in round |
| 6350-2.0 | 25.2 | Duration to complete alphanumeric path (trail #2) |
| 399-2.3 | 24.5 | Number of incorrect matches in round |
| 4283-2.0 | 24.2 | Number of rounds of numeric memory test performed |
| 4250-2.6 | 20.2 | Number of digits to be memorised/recalled |
| ***Lifestyle/environment alcohol*** | | |
| *No hits* |  |  |
| ***Lifestyle/environment tobacco*** | | |
| 20116-2.0 | 5.0 | Smoking status |
| ***Lifestyle/environment general*** | | |
| 20075-2.0 | 256.5 | Home location at assessment - north co-ordinate (rounded) |
| 4-2.0 | 166.4 | Biometrics duration |
| 5-2.0 | 105.3 | Sample collection duration |
| 1797-2.0 | 87.9 | Father still alive |
| 680-2.0 | 82.0 | Own or rent accommodation lived in |
| 738-2.0 | 79.0 | Average total household income before tax |
| 6142-2.0 | 76.5 | Current employment status |
| 1835-2.0 | 47.1 | Mother still alive |
| 709-2.0 | 42.6 | Number in household |
| 6138-2.0 | 38.5 | Qualifications |
| ***Lifestyle/environment exercise/work*** | | |
| 1070-2.0 | 31.9 | Time spent watching television (TV) |
| 6162-2.0 | 22.2 | Types of transport used (excluding work) |
| 1050-2.0 | 17.6 | Time spend outdoors in summer |
| 1130-2.0 | 12.7 | Hands-free device/speakerphone use with mobile phone in last 3 month |
| 6162-2.1 | 12.2 | Types of transport used (excluding work) |
| 1090-2.0 | 11.8 | Time spent driving |
| 924-2.0 | 10.3 | Usual walking pace |
| 981-2.0 | 9.0 | Duration walking for pleasure |
| 1011-2.0 | 8.8 | Frequency of light DIY in last 4 weeks |
| ***Mental health*** | | |
| 4537-2.0 | 21.2 | Work/job satisfaction |
| 4581-2.0 | 12.6 | Financial situation satisfaction |
| 2080-2.0 | 11.5 | Frequency of tiredness / lethargy in last 2 weeks |
| 4653-2.0 | 9.2 | Ever highly irritable/argumentative for 2 days |
| 2060-2.0 | 8.2 | Frequency of unenthusiasm / disinterest in last 2 weeks |
| 4570-2.0 | 7.8 | Friendships satisfaction |
| 1950-2.0 | 7.8 | Sensitivity / hurt feelings |
| 4526-2.0 | 6.9 | Happiness |
| 2090-2.0 | 5.4 | Seen doctor (GP) for nerves, anxiety, tension or depression |
| 2050-2.0 | 5.1 | Frequency of depressed mood in last 2 weeks |

*Table 1: Top 10 associations between non-imaging derived phenotypes (nIDPs) and standard principal components, grouped per category. The p-values reported in the table are the maximum p-values across the first two components. Only associations surviving Bonferroni correction across nIDPs and components are reported.*

**References**

[1] C. J. Fraza, R. Dinga, C. F. Beckmann, and A. F. Marquand, ‘Warped Bayesian linear regression for normative modelling of big data’, *Neuroimage*, vol. 245, p. 118715, Dec. 2021, doi: 10.1016/j.neuroimage.2021.118715.

[2] S. Rutherford *et al.*, ‘Charting brain growth and aging at high spatial precision’, *eLife*, vol. 11, p. e72904, Feb. 2022, doi: 10.7554/eLife.72904.

[3] R. Dinga, C. J. Fraza, J. M. M. Bayer, S. M. Kia, C. F. Beckmann, and A. F. Marquand, ‘Normative modeling of neuroimaging data using generalized additive models of location scale and shape’, Jun. 14, 2021, *bioRxiv*. doi: 10.1101/2021.06.14.448106.

[4] S. M. Kia *et al.*, ‘Closing the life-cycle of normative modeling using federated hierarchical Bayesian regression’, *PLoS ONE*, vol. 17, no. 12, p. e0278776, Dec. 2022, doi: 10.1371/journal.pone.0278776.

[5] A. F. Marquand, I. Rezek, J. Buitelaar, and C. F. Beckmann, ‘Understanding Heterogeneity in Clinical Cohorts Using Normative Models: Beyond Case-Control Studies’, *Biol Psychiatry*, vol. 80, no. 7, pp. 552–561, Oct. 2016, doi: 10.1016/j.biopsych.2015.12.023.

[6] T. Wolfers *et al.*, ‘Mapping the Heterogeneous Phenotype of Schizophrenia and Bipolar Disorder Using Normative Models’, *JAMA Psychiatry*, vol. 75, no. 11, pp. 1146–1155, Nov. 2018, doi: 10.1001/jamapsychiatry.2018.2467.
